# Young People’s Attitude Toward Positive Psychology Interventions: Thematic Analysis

**DOI:** 10.2196/21145

**Published:** 2020-11-09

**Authors:** Toni Michel, Franziska Tachtler, Petr Slovak, Geraldine Fitzpatrick

**Affiliations:** 1 Technical University Vienna, Wien Vienna Austria; 2 King's College London London United Kingdom

**Keywords:** adolescent, mental health, health resources

## Abstract

**Background:**

Digital instantiations of positive psychology intervention (PPI) principles have been proposed to combat the current global youth mental health crisis; however, young people are largely not engaging with available resources.

**Objective:**

The aim of this study is to explore young people’s attitudes toward various PPI principles to find ways of making digital instantiations of them more engaging.

**Methods:**

We conducted an explorative workshop with 30 young people (aged 16-21 years). They rated and reviewed 29 common PPIs. Ratings and recorded discussions were analyzed using thematic analysis.

**Results:**

Some interventions were conflicting with young people’s values or perceived as too difficult. Participants responded positively to interventions that fit them personally and allowed them to use their strengths.

**Conclusions:**

Values, context, strengths, and other personal factors are entangled with young people’s attitudes toward digital instantiations of PPI principles.

## Introduction

### Background

We are in the midst of an escalating global youth mental health crisis [1]. Among all age groups, young people are the most likely to develop mental health problems and the least likely to have access to support [2]. Suicide has become the most common cause of death for boys and second most common for girls aged between 15 and 19 years [3]. The majority of young people do not have sufficient access to mental health support [4]. Many who endure unresolved youth mental health problems consequently deal with them for the rest of their lives [5].

Thus, mental health promotion needs to be more widely available. Research has indicated that the most efficient way of confronting the crisis is by addressing mental health on a population level, as opposed to individual treatment, and that population-level mental health is best improved by expanding mental health promotion services [2].

Positive psychology (PP) is the area of mental health research oriented toward mental health promotion. According to Seligman and Csikszentmihalyi [6], “PP is the scientific study of positive human functioning and flourishing on multiple levels that include the biological, personal, relational, institutional, cultural, and global dimensions of life.” PP is thus complementary to the more conventional disease model of mental health, which is oriented toward resolving mental health problems [7]. The main goal of PP is to improve on positive aspects of mental health, such as well-being and optimism, grounded in the assumption that all human beings have the capacity to flourish, and existing strengths. Theoretically, PP is thus a continuation of humanistic psychology [8] and, more specifically, Maslow’s [9] notions of *health and growth psychology* in contrast to *low ceiling psychology*. In terms of intervention mechanisms, PP is a growing ensemble of diverse evidence-based interventions, which we call positive psychology interventions (PPIs). PPIs are often low in complexity and do not require excessive amounts of time or expert supervision [10]. These are often shared with common *intervention principles* such as forgiveness, mindfulness, and gratitude. Gratitude interventions, for example, have shown to improve life satisfaction, well-being, and positive affect and decrease negative affect [11,12]. To illustrate, one common intervention utilizing the gratitude principles is the *Gratitude Letter*, a reflective writing activity that consists of first writing a letter about all the things a person is grateful for toward another person and then delivering this letter [13]. According to a report from the Lancet Commission on Global Mental Health and Sustainable Development, digital instantiations of existing intervention principles are the most promising avenue for making mental health interventions (including PPI) available to young people because of the wide proliferation of digital devices and the (in principle) ease of scaling up digital solutions for large audiences [14]. Consequently, youth health care providers around the world have started offering them [15,16].

However, despite these ongoing efforts, and despite young people *reporting* interest in using digital instantiations of interventions, young people engage with such digital instantiations only to a limited degree: research revealed low uptake, low adherence, and low engagement of young people with digital mental health promotion [17,18]. A series of focus groups with young people (aged 15-16 years), which explored preferences in relation to mental health promotion apps, highlighted that “content should be made fun and interactive through the use of pictures, music, videos and games” [19]. Subsequently, another group of researchers investigated the degree to which the content of mental health promotion apps aligned with young people’s media preferences, discovering an overreliance on static content, that is, written text, and recommended more visual and interactive solutions [20]. Existing research did not investigate the degree to which these issues are a consequence of how PPI principles are translated for digital platforms, as opposed to being rooted in the PPI principles themselves.

### Objectives

To explore this further, we conducted a workshop on young people’s attitudes toward PPI and their underlying intervention principles, with 30 young people aged 16-21 years. We decided on the upper end of the *young people* age range because it allowed us to have more in-depth discussions, as would otherwise potentially have been the case.

**Figure 1:**
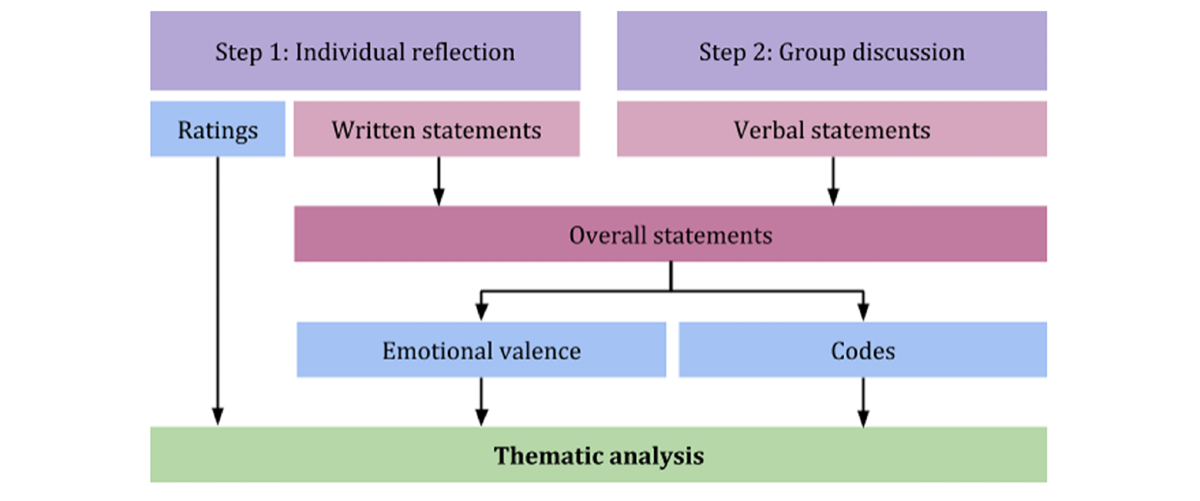
Overview of the 2-step research phase.

The research phase consisted of 2 steps: (1) *individual reflection* and (2) *group discussion*. During step 1, young people individually read, rated, and provided written statements on instructions for 29 common PPIs (refer to the *Methods* section for how we selected them). They received descriptions of the interventions, on paper, alongside a series of Likert scale questions to what degree the interventions fit to them personally and a textbox to express their thoughts in more detail. During step 2, they discussed the interventions in groups of 6. Both written and verbal statements were subsequently categorized by emotional valence, that is, as positive, balanced, or negative and coded. We assessed valence to allow a systematic overview of how positive, neutral, or negatively specific PPI were perceived. All collected data informed the construction of themes according to thematic analysis [21]. An overview of the study process is shown in Figure 1.

We will differentiate between specific PPIs (eg, *Gratitude Letter*), the intervention principles (eg, *Gratitude*), and their digital instantiations (eg, an app with which to write a *Gratitude Letter*). The differentiation between interventions, intervention principles, and digital instantiations aligns with the recent calls to focus on the principles more than specific interventions to produce long-term, reliable insights [22].

When analyzing our data, we discovered that the criticisms young people had *for the intervention principles* were similar to the issues that previous research had attributed *to digital translation of the existing PPI*. In other words, a common assumption in digital mental health research has been that—as research provides us with evidence-based interventions—it *only* requires engaging reinterpretations of interventions and (young) people would be interested in them. If the issue with nonengagement was rooted in how we translate interventions for digital platforms, then we would not expect to find the same criticism leveled against conventional, nondigital interventions. However, the findings of this study indicate that the issues young people see in digital instantiations of PPI are in fact *inherited* from their nondigital predecessors. We might not just need to improve the quality of digital interpretations to make them more engaging for young people but might need to reevaluate the appropriateness of their underlying intervention principles.

This paper is structured as follows. We first describe how we recruited 30 young people from a German school to participate in the workshop. We then explain how questionnaires and audio recording during the workshop were used for data collection, followed by how we analyzed the data using common statistical methods and thematic analysis. We then discuss the wider implications of our findings for the design of digital interpretations of PPI for young people.

## Methods

### Context

This study is part of a project that investigates how technology may be applied for mental health prevention and promotion in young people, especially how digital instantiations may be designed to be engaging for young people. Insights generated from this study will facilitate applied design research, that is, the creation of a digital toolkit to support prevention approaches for young people.

### Selection of Methods and Setting

We decided to conduct a workshop with young people for the following reasons. Our goal was to investigate young people’s attitude toward PPI; thus, young people would be our participants. Our questions were explorative and open ended; thus, they would best be addressed by predominantly qualitative data. We wanted our participants to speak openly and potentially critically about the subject matter; thus, they should discuss with their peers, reducing the impact of power dynamics between researchers and participants. Finally, we wanted to set up our research in a way that would provide benefits for our participants. Utilizing the multidisciplinary background of our research team, consisting of experts for design, technology, and mental health, we developed a workshop for designing digital instantiations. This workshop explored young people’s attitude toward PPI and allowed them to design their own digital instantiations of intervention principles by learning to apply common industry design and design research methods, such as mood boards, personas, and low fidelity (ie, paper-based) prototypes. As a location for the workshop, we decided on a rural German high school with a specialization for art and design. The school we chose prepares students for higher education in artistic disciplines, anticipating they would act as creative design partners (as they did). Methods we taught during the workshop were discussed before with local teachers to make them suitable for the curriculum.

### Ethics Approval and Proceedings

We first contacted the Department of Science and Education for the State of Saxony in Germany to inquire about the protocol for conducting research in local schools. They provided us with a series of questionnaires aimed at identifying possible harm and possible benefits for students, which could result from our research. On the basis of our answers, we were given permission to proceed with the study. We then contacted a local school. The school administration allowed the workshop to proceed. We then presented the plans for our workshop to the students. The school administration recommended a specific class for which they assessed the most potential benefit from attending the workshop.

The workshop took place over 3 days before the start of a school holiday. Students of this class were given the choice of attending the workshop or continuing with their regular lessons. All 30 students opted to attend the workshop. As part of enrolling them into the study, every participant received information about the workshop, had the opportunity to ask questions, was informed about their rights as participants, and subsequently signed an informed consent form. For those aged under 18 years, we also required written consent from their legal guardians. All documents were presented and collected during the workshop. Regarding data collection, we decided against video recording of group discussions because of privacy concerns, given the sensitivity of the topic, that is, mental health, and the nature of our participants, that is, young people (a trade-off, which meant that it was not possible to link participants and quotes during data analysis).

### Participants

Our workshop had 30 participants, aged 16 to 21 years, with a median age of 18 years and variance of 1.9 years. A total of 50% (15/30) of our participants identified as female, 43% (13/30) identified as male, 3% (1/30) identified as nonbinary, and 3% (1/30) did not self-identify their gender. In terms of nationality, 70% (21/30) identified as German, 10% (3/30) identified as Polish, 3% (1/30) identified as Serbian, 3% (1/30) identified as Taiwanese, 3% (1/30) identified as Malaysian, 3% (1/30) identified as Turkish, and 3% (1/30) identified as Chinese.

### Procedure

The workshop was conducted over the course of 3 days and consisted of a research phase and a design phase. The research phase is the focus of this paper and consists mainly of a 120-min long 2-step activity, that is, individual reflection and group discussion. During the subsequent design phase (not a subject of this study), the participants went on to create their own concepts for digital instantiations of PPI principles. The workshop took place in a classroom with nonmovable desks and a maximum capacity of 50. Timeline of the workshop is given in Table 1, and an overview of the general structure is shown in Figure 1. The workshop was moderated by one researcher from our group.

**Table 1:** Timeline of the workshop.

Time	Step	Description
10 AM to 10:15 AM	Introduction	Participants were reminded of the workshop’s purpose and informed about the overall structure. They then received a brief introduction to PP^a^ to improve their ability to process the later stages. At the end of the introduction, the participants were randomly assigned to 5 groups of 6 participants each.
10:15 AM to 10:45 AM	Step 1: individual reflection	Participants received 4 to 5 descriptions of PPI^b^ (“Intervention sheets”) so that each group had intervention sheets for all 29 interventions, randomly assigned to participants within groups. The general structure of the intervention sheets can be seen in Figure 2. Participants then individually read the intervention descriptions and answered 4 questions about them: “How likely would it be that you try this intervention?” “How well does this intervention fit to you?” “How much would you expect this intervention to help you?” “Why?” Questions (1) to (3) were rated on a 5-point Likert scale, with 1=“not at all” and 5=“a lot.” Question (4) was answered in writing with space for 2 to 3 sentences (some participants opted to write longer answers on the backside of the sheet).
10:45 AM to 11 AM	Break	N/A^c^
11 AM to noon	Step 2: group discussion	Participants were asked to discuss within their group how much merit they saw in each intervention. This consisted of participants first presenting to each other the interventions they reviewed during step 1 and then discussing them. These discussions were audio recorded through recording devices placed at the center of each group’s table. A moderator familiar with PP was available to answer the questions in case the descriptions were unclear.

^a^PP: positive psychology.

^b^PPI: positive psychology intervention.

^c^N/A: not applicable.

**Figure 2:**
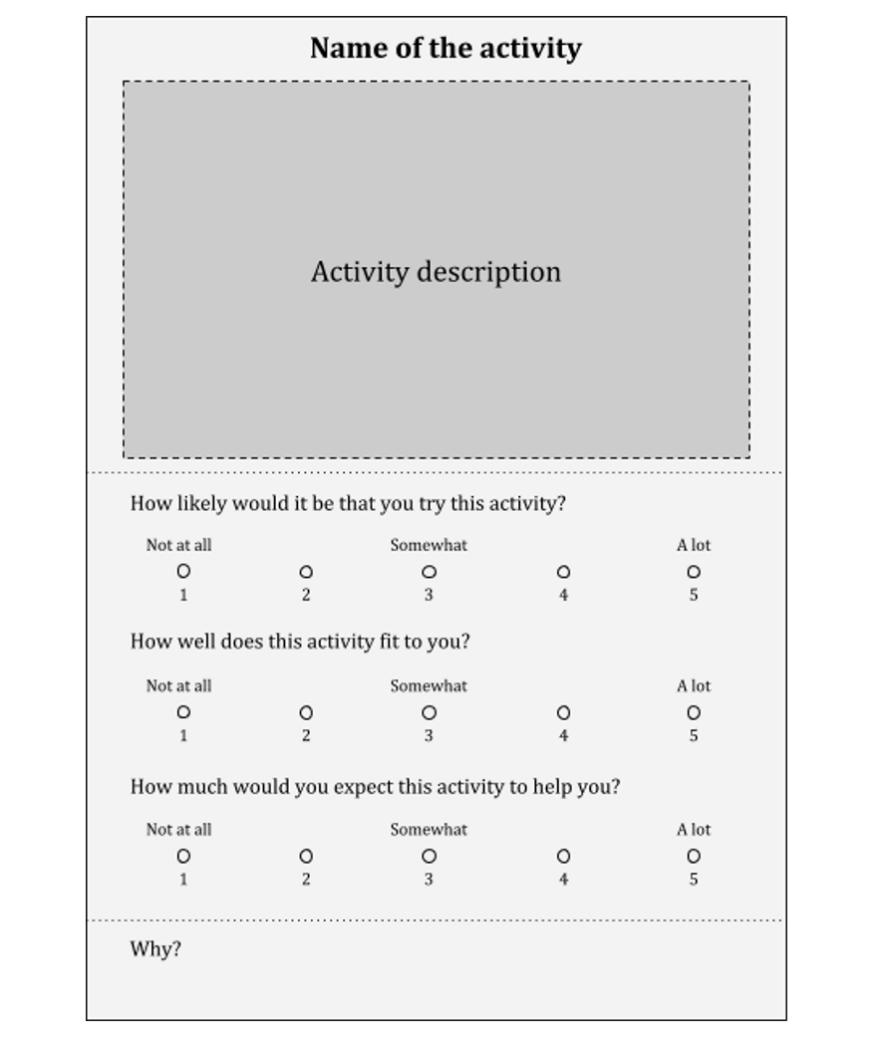
Intervention sheet template.

### Interventions

To assemble a suitable selection of interventions, we consulted a web-based database for mental health experts, the Positive Psychology Toolkit [23]. To our knowledge, at present, the platform is the most comprehensive PP database. In addition, it allowed us to search by intervention principles, that is, family of related intervention strategies [24], for example, *Mindfulness* and *Gratitude*, which was useful when looking for representative PPI from common intervention strategies. We selected interventions based on the following criteria, which we derived from a discussion within our research group:

#### Youth Appropriateness

The interventions should be applicable to school-aged young people, that is, not explicitly reference *offices*, *coworkers*, *retirement*, or other not age-appropriate concerns, to maximize the likelihood that the interventions would align with the lived experiences of our participants.

#### Evidence Based

The interventions should be evidence based, that is, based on peer-reviewed research, to exclude interventions that have not yet been shown to provide benefits.

#### Easy Overview

We excluded *complex* interventions that consisted of multiple phases or sessions or multiweek programs, to have descriptions that would be accessible to our participants. Given that there would be limited time during the workshop for our participants to understand how these interventions worked and that our participants did not have professional mental health backgrounds, we focused on interventions with few steps.

#### Manageable Amount

We estimated that between 25 and 30 interventions would be ideal, to keep the overall number of interventions low enough so our participants would be able to briefly discuss all of them during a 60-min group discussion. We selected them from 315 PPIs available in the database.

#### Diverse Content

We included diverse kinds of interventions, spread across domains (eg, communication, happiness, and mindfulness) of varying duration (5- to 60-min interventions) and using a variety of modalities (eg, writing, photos, music, and physical intervention), to prompt a diverse range of responses.

After working through the database and applying our criteria, we arrived at a list of 29 interventions that were considered suitable for the context of the workshop (Table 2). This process was subjective; other researchers may have included some interventions we dismissed and vice versa, especially concerning youth appropriateness and diverse content. However, although an objective selection was not possible, we opted for *intersubjectivity* through consensus within our group and discussed extensively which PPI to include. We apply *intersubjectivity* in the sense of Heidegger’s use of the term [25]. As there was no objective answer about which interventions to select, we instead aimed for agreement between subjective opinions within our research group.

The interventions offered by the Positive Psychology Toolkit and PP research in general are strongly influenced by Western cultures, in particular US culture. Descriptions of interventions were entirely in English, so was the underlying research, and so was (likely) the native language of most participants of the studies on which the interventions were originally validated. For our context, that is, young people from Germany, one of the researchers in our group translated the intervention descriptions into German and into a reading level that was appropriate for our participants, for example, removing technical jargons that would not be familiar to readers without a background in mental health. However, cultural assumptions and values within the interventions could not be *localized*, and it is possible that these cultural assumptions and values have had an impact on our participants’ responses.

**Table 2:** Interventions.

ID	Name	Domain	Time (min)
1	Apologizing Effectively	Communication	5
25	Three Loving Connections	Communication	5
17	Nonjudgmental Reflection	Compassion	15
20	Reframing Critical Self-Talk	Compassion	10
21	Self-Compassion Break	Compassion	5
12	Healing Through Writing	Coping	15
15	Initiating Physical Activity	Coping	30
18	Positive Emotion Brainstorm	Emotions	15
26	Using Music to Express Feelings	Emotions	N/A^a^
13	Hope Map	Goals	30
22	Self-Contract	Goals	10
6	Gratitude by Mental Elimination	Gratitude	15
7	Gratitude for Important People	Gratitude	10
8	Gratitude Journal	Gratitude	10
9	Gratitude Letter	Gratitude	20
10	Gratitude Meditation	Gratitude	10
14	Increasing Awareness of Complaining	Gratitude	N/A
2	Chasing Happiness	Happiness	5
3	Creating Flow Experiences	Happiness	45
11	Have-a-Good-Day Exercise	Happiness	10
19	Random Acts of Kindness	Happiness	N/A
23	Spending Money on Others	Happiness	N/A
28	Writing About Intensely Positive Experiences	Happiness	10
4	Creating Quiet Time	Mindfulness	5
24	The Best Possible Self	Mindset	10
5	Daily Motivational Awareness	Motivation	5
27	Using Photography to Increase Savoring	Savoring	10
29	You, At Your Best	Strengths	60
16	My Gravestone	Values	12

^a^N/A: not applicable.

### Data Analysis

We collected 146 written responses to interventions from step 1, that is, overall 146 statements the participants wrote on the same sheet of paper on which they rated the interventions. The transcribed group discussions from step 2 resulted in an additional 220 statements, that is, 366 statements overall. We labeled a *statement* any comment that related to how PPIs were perceived; we excluded the descriptions of PPIs and off-topic conversations. Alongside the statements, we also collected Likert scale ratings for all PPIs, which participants made individually during step 1.

Subsequently, thematic analysis [21] was led by one of the researchers, after which the statements were translated into English by a German native speaker (so as to be easily processed by our mostly English-speaking team). The researcher started by reading the statements without initial codes. They wrote down candidate codes while working through the statements, based on what they perceived as the best characterization of the respective statements. After working through all the statements, they started again from the beginning, reapplying codes from the first reading, while extending them with new codes and merging and splitting codes based on further reflection. In addition, the statements’ emotional valence was classified as either *positive*, that is, the participant seemed to like the PPI (eg, “What I like about this intervention is...”); *balanced*, that is, it was not clear whether the participant liked the PPI or not (eg, “I don’t know what to think of this intervention”); or *negative,* that is, the participant seemed to dislike the activity (eg, “What I don’t like about this intervention is...”). This process was concluded after the fourth iteration because during this iteration, no further changes to the codes seemed necessary. The codes were then written out on post-it notes and grouped by perceived thematic similarities. Over the course of 1 week, codes were moved through different group configurations in search for what seemed their most meaningful combination. The process involved 4 researchers from our group and was accompanied by many discussions to reduce bias.

The Likert scale ratings from step 1 and the emotional valence of statements (positive, balanced, or negative) served for additional sense checking. Likert scale ratings were processed by calculating the median rating for each PPI and correlated with certain strategies (eg, *Mindfulness*). We identified 3 major clusters, from which the themes were constructed. We deliberately frame this process as a *construction*, as we follow Braun and Clarke’s [21] position that themes do not reside within codes or are discovered. Instead, they result from the active decisions of researchers about what to emphasize and what to deemphasize while purposefully constructing a meaningful narrative [21].

## Results

### PPIs

Step 1 produced 145 ratings (next to 146 written statements)—5 ratings for each of the 29 PPI—consisting of 3 components (corresponding to the 3 Likert scale questions on the score cards): *inclination* (of participants to try the PPI), *expectation* (of participants that the PPI would help them), and *fit* (to our participants interests). Although there was notable variation in ratings between PPI, within PPI, the 3 aspects (inclination, expectation, and fit) were consistently close (a plot of the data is shown in Multimedia Appendix 1). This suggests that inclination, expectation, and fit were, to our participants, related, although further statistical analysis based on a larger sample would be necessary to make this point with higher confidence. Across all ratings, if a participant gave a low rating for fit, he or she would also give a low rating for expectation and inclination. The same applies to high ratings.

**Figure 3:**
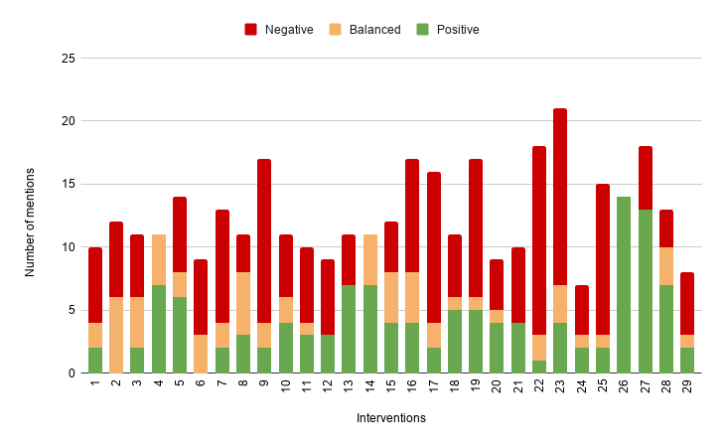
Emotional valence expressed toward positive psychology intervention.

By combining 146 written statements from step 1 and 220 group discussion statements from step 2, we arrived at 366 unique statements participants made toward the 29 interventions. As explained in the *Data Analysis* section, we coded each statement and categorized the emotional valence of statements as either positive, balanced, or negative. Of the 366 statements, 33.1% (121/366) were positive, 16.7% (61/366) were balanced, and 50.3% (181/366) were negative. An overview of emotional valence expressed toward PPI is shown in Figure 3.

### Themes

#### Construction of Themes

We constructed 3 themes from step 1 (ie, individual reflection) and step 2 (ie, group discussion). The rating of step 1 served for additional sensemaking, that is, when deciding whether a candidate subtheme sufficiently reflected trends or majority of opinions during the workshop. For example, if one group would have discussed a certain PPI largely negatively, but the overall rating for this PPI was positive, then we would take this discrepancy into consideration. Practically, the ratings largely aligned with the qualitative statements and thus gave us additional confidence about the appropriateness of subsequent themes. The quotes we decided to highlight are canonical examples of common statements during the workshop.

The first theme deals with points of tension between youth cultures and values embedded in PPIs. The second theme addresses the possibility of PPI failing and how it may impact young people. The third theme is concerned with the impact of individual differences and preferences on our participants’ attitude toward PPI.

#### Theme 1: Youth Cultures

It became evident during the discussions that PPIs have a normative layer and reflect assumptions about desirable behaviors. Strategies involving, for example, gratitude and forgiveness are not morally neutral; instead, they have wrapped into them assumptions about what behaviors are *good* and that lack thereof is thus *bad*. Forgiveness, for example, may be viewed as something positive within the moral system within which PP is situated but has also been described as a trait symbolic of weakness [26]. These moral assumptions created tensions to the degree that they were perceived as diverging from the values and norms of our participants, that is, their cultures. Especially notable were tensions around the areas of (1) *inauthenticity*, (2) *docility*, (3) *pathologization*, and (4) *appropriateness*.

(1) *Inauthenticity*: Our participants were critical of interventions when they pushed toward behaviors that would not have occurred *naturally*. Performing certain behavior against one’s inclinations was perceived as dishonest. These types of interventions triggered terms related to oppression, such as *forcing*, *dictating*, and *pushing* (Q1.1 to Q1.3).

Q1.1: Random Acts of Kindness:

I think you ought to be friendly to other people because you like them, and because you yourself are a friendly person, otherwise you force yourself into a shape that is not really you.

Q1.2*:* Three Loving Connections:

I think you should spend time with people because you yourself have a personal need for that and not because some plan dictates that you should.

Q1.3*:* Gratitude Letter:

For me this definitely wouldn’t work, because, I would feel pushed to write a letter just to make the other person happy, not to actually reflect on the things I’m grateful for.

(2) *Docility*: A range of interventions was criticized for promoting docile behavior. Our participants spoke negatively about interventions that recommended apologizing and being grateful (Q2.1 to Q2.3). Although they considered that some of these behaviors may be sensible in a measured approach, they worried about people being encouraged to lean toward these behaviors beyond a healthy degree (Q2.1). They also perceived some interventions as accusatory because the intervention sounded to them as if they are not already, for example, apologizing enough (Q2.2).

Q2.1: Apologizing Effectively:

The thing is, when I don’t have confidence anyway, and then I’m expected to apologize for everything, doesn’t that do more harm than good? —Seriously, maybe I apologize already too much. Like, they think you’re a completely egotistical person and that you wouldn’t apologize by yourself. (...) For me, this wouldn’t work.

Q2.2: Gratitude Letter:

I also see a risk in that. Here, with all these Gratitude things. They say, ok, write up what you are grateful for. And if you can’t think of anything, are you then un-grateful? But what if there actually are no things to be grateful for? Sometimes you just feel bad, sometimes you get treated badly, and then you are supposed to be grateful. It’s like, eat shit and smile. Do you know what I mean? Maybe it needs an un-grateful letter—yeah, to emphasize—to just emphasize all the things that hurt, or that did hurt, or cause problems—and to show that to someone. You hurt me, and I’m not fucking grateful. It’s also easy to say what you are grateful for because people want to hear that, there is no barrier. Telling someone, this hurt me, you were unfair to me, you caused me problems, that is much more difficult. And if it’s then someone who is above you, like a teacher—yeah you may be laughing but I really could write an un-grateful letter like that to one or two teachers here—but then I only get in trouble.

Q2.3: Gratitude for Important People:

This is like something—I used to go to church as a kid, I had to, and this sounds incredibly preachy, like something my pastor would tell me to do. Maybe this means I’m a bad person, but I actually don’t think I need to act like that.

(3) *Pathologization*: Our participants expressed several times that perceived *negative* behavior may still lie within the range of healthy functioning and that it does not necessarily need to be corrected (Q3.1 to Q3.3). This also intersected with triggers of negative emotions for some, for example, regarding sports and weight loss (Q3.2). Triggering negative emotions is discussed more in theme 2. It was questioned whether happiness *all the time* is necessary and said that being happy *wouldn’t mean anything* if its rooted in superficial reasons, such as *antidepressants—*a category within which the participant seemingly also placed PPI (Q3.3). (To note, this criticism may be rooted in us not sufficiently having explained to our participants the difference between hedonic and eudemonic happiness and PP’s orientation toward the latter).

Q3.1: Random Acts of Kindness:

You may be mentally entirely healthy, and everything is fine and you’re still just an asshole. Being an asshole doesn’t mean you’re sick or need therapy, it only means you’re an asshole.

Q3.2: Initiating Physical Activity:

I know I’m a bit overweight, but that also means that I hear all the time that I’m supposed to do more sports. Weight loss may not be what they have in mind with this intervention, but the topic is still a major trigger for me. Some people just do not like to do sports. It’s ok not to like sports. You don’t need to do sports to be a normal healthy person.

Q3.3: Chasing Happiness:

Must I feel happy all the time? They seem to assume—if you aren’t happy then something surely is wrong with you, so you better try to be happier. Right? It may sound strange, but I think its ok that I don’t feel happy all the time. I may not even feel happy most of the time. If I wanted to feel happy all the time, I could just gobble up some anti-depressant meds, but that wouldn’t really mean anything, would it?

(4) *Appropriateness*: Our participants suggested that the appropriateness of a PPI is bound by its context and that context could provide an anchor to normalize an intervention (Q4.2 to Q4.3). Some interventions made more sense to our participants when placed in relation to special times. Although, for example, gift giving without a contextual anchor could appear strange; the birthday of a friend could constitute such an anchor and thus normalize the intervention (Q4.2). Similarly, one participant stated she would feel uncomfortable if given a Gratitude Letter *out of the blue*, that is, lacking a contextual anchor, and that she would feel *disturbed* by it (Q4.1).

Q4.1: Gratitude Letter:

Imagine you get a letter like that—I’m just saying, out of the blue a buddy walks up to you and gives you one of those letters, I’d be seriously wondering what the hell went wrong with him. I’m always there for my friends, obviously, and sure I’m happy about the occasional “thanks,” but if someone would go through the trouble of writing a letter like that, I probably wouldn’t feel happy, more disturbed.

Q4.2: Spending Money on Others:

A friend of mine recently celebrated her birthday—I was feeling a bit down at the time, but I got up and got her movie tickets—and I noticed that when I gave them to her, I actually felt better. I thought that was a bit odd at the time, but now I read this, and I think, yeah, that does make perfect sense to me.

Q4.3: You, At Your Best:

Yeah, so, with this intervention, “You, at your best,” I actually did something like this a while back. When I finished middle school, I noticed that in many ways I wasn’t really happy with myself. So, before I started here, I sat down at home, and just sketched out how I wanted to be—probably didn’t take as much time for it as they recommend here, but essentially the same thing.

In summary, our participants highlighted a series of issues related to tensions between their personal values and norms and those imbued in PPI. Culture and context seemed to play relevant roles in determining when an intervention would be appropriate. Wider implications of this theme are discussed in the *Discussion* section.

#### Theme 2: Conditions and Consequences of Failure

Our participants were concerned that many interventions harbored the potential to fail and how they would deal with the setback if that would happen. Related to this, they expressed concerns over PPI inadvertently, making them feel worse instead of better. This theme is structured into the subthemes (1) *intrinsic factors,* (2) *extrinsic factors,* and (3) *triggering of negative emotions.*

(1) *Intrinsic factors*: Participants pointed out an inherent degree of difficulty of some PPIs (Q1.1 to Q1.2). They attributed this difficulty to either the intervention itself (Q1.1), own perceived shortcomings (Q1.2), or a lack of experience with PPIs (Q1.3). One participant compared PPIs with yoga exercises, in that experience is necessary for exercises to become easier but that first starting is the most difficult step (Q1.3).

Q1.1: Creating Flow Experiences:

I also think this is difficult—this is complicated, and not necessarily something that you can do quickly. You first must understand all the steps. You almost have to study in order to understand this.

Q1.2: Nonjudgmental Reflection:

It would be difficult for me to not have judgmental thoughts.

Q1.3: Gratitude Meditation:

I mean, I’ve never done anything like this, maybe for someone who does nothing but meditating from dusk ‘til dawn—maybe then that’s easy, but seriously—do you understand this? This may be something like yoga, where you get better at doing the poses over time, but it’s really damn difficult to get started.

(2) *Extrinsic factors:* This subtheme relates to difficulties that are not necessarily rooted in the intervention but in some contingent elements, for example, how other people involved in the intervention may respond (Q2.1 and Q2.3) or potentially falling short of overambitious goals (Q2.2). One participant emphasized that getting a positive response from the receiver of a gift during the Spending Money on Others intervention “may actually make you feel better” (it does [24]) but that it still involved a risk of rejection (Q2.1). Risk of rejection also came up during another participant’s response to Three Loving Connections, asking what should be done “if the people don’t want to meet you though” (Q2.3). Another participant reflected on negative experiences with the New Year’s resolutions, which she likened to the Self-Contract PPI and raised the issue of what to do if one falls short of their own set goals (Q2.2).

Q2.1: Spending Money on Others:

If you get the wrong response after giving someone a gift then that doesn’t really help you. But if you get a positive response—A positive response may actually make you feel better, but it could end up being disappointing otherwise.

Q2.2: Self-Contract:

This “self-contract” thing sounds like new year’s resolutions to me—I’ve been through that with my dad. We planned which grade I should reach in which subject, and then that didn’t work out. And then that was just really depressing. So, what happens if I can’t fulfill the contract? Then I’m just standing there, disappointed.

Q2.3: Three Loving Connections:

For me, I’d be worried about getting rejected, if I’m honest. I may not be the most social person, and in principle that’s ok for me, but if the goal of this intervention is to meet more people—it says nothing what to do if the people don’t want to meet you though.

(3) *Triggering negative emotions*: This subtheme relates to PPIs being a potential cause, that is, trigger, for negative emotions. Both a focus on negative (Q3.1) and positive (Q3.2) memories could, according to our participants, become a trigger for them. Additional risk was identified in relation to Daily Motivational Awareness, an activity that asks people to reflect on what drives them. One of our participants worried how to reconcile if *nothing* motivated her, saying that “realizing that would be really depressing for me” (Q3.3). Explicit reference to a PPI being *depressing* also came up in response to My Gravestone, where people are tasked to reflect on what they would want to be remembered for. The participant noted that this PPI would “put all the focus on dying,” saying that they “don’t want to think about that” (Q3.4).

Q3.1: Healing Through Writing:

What hurt me? That sounds contra-productive. You are supposed to really think deeply about something that hurt you, and really immerse yourself in that pain? Fuck that shit. I don’t want that—I really don’t want to put a magnifying glass on that. If someone would say, ok, please write this down, then I’d say, piss off, easy as that.

Q3.2: The Best Possible Self:

I’m just feeling that my memory of my “best self” from the past could lead to the thought that this good time is now over and that I’ve lost something positive. If the response to that would be a clinging to the past, then I don’t think that would be good.

Q3.3: Daily Motivational Awareness:

What if nothing motivates me? So, what if I do things only because I must do them, but beyond that I cannot find a reason for it? Realizing that would be really depressing for me.

Q3.4: My Gravestone:

It’s as depressing as it sounds. You put all the focus on dying. I don’t want to think about that. I mean, I understand—it’s supposed to create perspective, ok, but—what I would end up thinking about is dying—and that’s just depressing.

In summary, our participants discussed the risk of interventions failing them, either because the intervention itself may be too complicated or because other factors outside of the intervention may not manifest as they should. They also spoke about the issue of negative emotions being triggered by PPIs. Wider implications of this theme are discussed in the *Discussion* section.

#### Theme 3: The Impact of Personality and Modality

This theme deals with individual factors in relation to PPI, that is, factors that are highly subjective, differ between people, and are rooted in tastes and preferences. There are 2 subthemes: (1) *impact of personality differences* on PPIs and (2) *role of modalities* within those interventions.

(1) *Impact of personality differences:* Young people are no homogenous group with regards to their interests and preferences, and their individual differences impact how they respond to PPIs (Q1.1 to Q1.4). In some instances, this was explained with reference to personality traits, such as a tendency toward anxiety (Q1.1) or lack of patience (Q1.3). In other instances, participants just noted that an activity would be uninteresting for them, without going into details as to why (Q1.2). One participant who said she was too anxious for meditation referenced an alternative activity that would work better for her, playing soccer (Q1.1). Another participant reflected on a writing-based PPI, saying that she did not have “a talent to write stories like that” (Q1.3). Participants commented positively on PPI, which allowed them to use what they saw as personal strengths (Q1.4).

Q1.1: Gratitude Meditation:

I personally don’t think this intervention fits to me because I personally am too anxious for meditation. I mean, the goal is to relax, right? I can relax better when playing soccer, or something like that, afterwards I am calm, relaxed.

Q1.2: Nonjudgmental Reflection:

You know me (name redacted), I don’t need to try this to know that I wouldn’t enjoy this crap at all. Nothing about this is even remotely interesting for me.

Q1.3: You, At Your Best:

I didn’t particularly like this, because not everyone got a talent to write stories like that; I mean, to formulate this properly (...) I wouldn’t have the patience to sit down, pick something and write it out. For other people this could work, if they had the time and inclination, but for me, or even for most people maybe, this wouldn’t work.

Q1.4*:* Gratitude Journal:

Reading an intervention and seeing that it plays off something I’m already good at definitely makes it more likely for me to try it out. The intervention itself may already be difficult, without me having to deal with something I’m bad at on top of that. For example, I just don’t like to write, I’m not good at it, and it frustrates me. So why would I pick an intervention that forces me to write, instead of doing something I’m good at and enjoy?

(2) *Role of modalities*: Participants commented predominantly negatively on writing-based activities, stating that they would rather avoid this modality if possible (Q2.1 to Q2.2). Conversely, music (Q2.2) and photography (2.3) were commented on positively. In relation to music, one participant stated that “when (she) feels bad, (she) listens to music anyway” (Q2.2). In relation to photography, it seemed more important to one participant that there would be some visual options, “doesn’t matter if photos or videos, but something visual would be great for me” (Q2.3).

Q2.1: Reframing Critical Self-Talk:

What I like about this one—at least you do not have to write anything—always all this writing with these exercises. This shows, it doesn’t have to be writing all the time, you only need to think about it in this exercise, that’s much more pleasant—and if I want to write it down, then I can still do that.

Q2.2: Using Music to Express Feelings:

Finally, an intervention that fits to me—When I feel bad, I listen to music anyway—and usually it ends up helping me. I was asking myself when finally, an intervention comes up that I like, most things are just about writing.

Q2.3: Using Photography to Increase Savoring:

Photography would be fun for me—that seems like a great way to store positive memories—doesn’t matter if photos or videos, but something visual would be great for me.

In summary, attitude toward PPIs seemed predicated on personal preferences and strengths, partially in relation to personality and partially in relation to which modalities were dominant in a PPI. Writing components were especially criticized.

## Discussion

### Context

As described in the beginning, previous research had identified a lack of engagement from young people with digital instantiations of PPI and proposed to explain this through how PP principles are usually instantiated digitally, that is, with an overreliance on static context, such as written text. We investigated whether the lack of engagement of young people with digital instantiations may also be rooted in the underlying intervention principles, from which the digital instantiations PPI are derived. The findings of this study support this assumption; we will now discuss the findings in more detail.

### Principal Findings

Steps 1 and 2 of the workshop both explored participants’ attitude toward PPI, part of which was the quantitative rating of interventions and part of which were the qualitative statements about interventions. Both lines of inquiry converged toward the following notable insights.

Youth cultures may inform participants’ attitudes toward a PPI, which became visible when values and norms of our participants came into conflict with values and norms embedded in a PPI. Special concern was given to authenticity and a notable aversion toward PPIs that enforced docile behavior or that pathologized what our participants categorized as *normal* behavior. Our data suggest that young people should have a significant choice when it comes to when to apply which PPI, including how to shape the PPI to make it more appropriate for their context.

Young people may see PPIs as potential sources of negative emotions. If they feel that a PPI is too difficult—either inherently or made too difficult by too ambitious goals—then that may become a red flag that keeps young people from engaging with the activity. Further, components in some PPIs may become triggers for negative emotions, for example, a reflection PPI that may bring up negative memories. PPI instructions so far do not seem to take this possibility into account, at least not to a degree that our participants saw as sufficient. Research into how to safeguard PPIs from inadvertently becoming triggers for negative emotions—that is, which kinds of components most likely could become triggers—seems necessary. If we knew which interventions have a higher likelihood of triggering negative emotions, we could, for example, amend them with a suitable warning or not give them out to young people who are, at that point, not stable enough.

Individual preferences had a major impact on young people’s attitudes toward interventions. When talking about what they thought about interventions, our participants did so mostly in relation to their own preferences and interests. Although some preferences or aversions were more prevalent than others—for example, a general disdain for writing-based activities and preference for visual- and audio-based instantiations—there was an overall notable diversity in what young people felt appropriate for themselves. This diversity means that there cannot be a one-size-fits-all approach to youth mental health promotion. Instead, choice and customization may be the most relevant goals in any attempt to proliferate digital instantiations of PPI among young people. This may be especially relevant as the cultural context in which an intervention is first validated may not translate to another context, for example, Gratitude interventions have not yet been validated with German youth.

One promising way of approaching the complexities of individually varying interests and preferences may be found at the intersection of mental health and games research. Fleming et al [27] have developed a taxonomy of predispositions with which young people approach mental health tools, differentiating between *players or gamers*, that is, those from whom fun had the highest priority; *engagers*, that is, those for whom support to their well-being had the highest priority but who are open to gamified approaches; *skeptics*, that is, those who do not see value in digital interventions; and *Straight-talkers*, that is, those who explicitly do not want any gamified content in their mental health applications. Building on taxonomies like this would allow the creation of more focused strategies, such as how to best address *engagers*, instead of just aiming at *young people* in general.

Several issues brought up by our participants further intersect with ongoing discussions of PPI and underlying intervention principles; we have addressed these issues in the following sections.

### PPIs and Failure

Our participants were concerned about PPIs potentially making them feel worse, not better. Three possible reasons for this were given: the intervention itself being too difficult, for example, having too many steps; other people responding negatively, for example, an apology being rejected; and interventions triggering negative emotions, for example, by invoking problematic memories.

The literature on a PPI *backfiring*, as other researchers have called it [28], is sparse. Gratitude interventions were demonstrated to sometimes trigger feelings of “obligation, indebtedness, embarrassment, awkwardness and guilt” in a 2017 study in the United Kingdom [12]. There have been concerns that failing at a PPI may, for people with depression, result in a feeling of failure and give them “further evidence that they are defective” [28]. One PPI discussed during the workshop—*Healing Through Writing*—asks the person to reflect on a particularly painful memory. However, a 2009 study in the *Journal of Abnormal Child Psychology* has demonstrated that these types of memories are potential triggers for suicidal ideations in urban youth [29].

Beyond that, there is limited research on PPI *backfiring*. There are 2 possible reasons for this lack of research. First, it could be that PPI rarely go wrong and that our participants were overly cautious of something that in reality does not significantly factor into the application of PPI. If so, then our data at least suggest that the *concern* of PPIs *backfiring* is present among young people, which in turn means that PPI instructions should address it. Alternatively, the lack of research into PPI failing may also be an artifact of publication bias; it is possible that PPI studies that encounter significant issues only rarely make it to print. We are not able to say which of those is the case but suggest that it warrants further investigation.

### Faithfully Translating PPIs for Digital Platforms

The shortcomings of PPIs with regard to individual preferences and modalities line up with the aforementioned study on PP apps for young people [20], which showed an overreliance on static text and writing and a lack of customization for young people to shape interventions according to their own preferences and strengths, in contrast to what young people expect from digital instantiations [19]. The researchers attributed these issues to technology design, saying that “the design of these technologies needs to be more closely oriented to what young people are actually interested in” [20]. Although we do not disagree with this assessment, our study indicates that the issue is rooted in a deeper level. The overreliance on text and lack of customization may not per se be a consequence of an insufficiently ambitious design but instead may be a consequence of faithfully instantiating PPI principles into a digital context.

The impact of personal preferences on effectiveness is increasingly recognized in PP research. The Person-Activity Fit (PAF) model describes how the overlap between *person features*, such as preferences and strengths, and *intervention features*, such as dosage and variety, impact how well a PPI is performed, which in turn impacts the subsequent well-being increase [13]. Both previous research on digital instantiations of PPI principles for young people [20] and our research presented in this paper suggest that PAF between young people and PPI principles may be overall low, at least with regard to how PPI are currently being delivered, in both digital and analog formats.

### Design Implications

Instantiating conventionally text-based PPI for a digital platform could mean an opportunity to reinterpret these interventions to better fit with expectations and needs of young people across various cultures and subcultures. Activities in conventional PPI are mostly predicated on paper, pens, and the material constrictions they come with. For example, a conventional diary entry would not allow a young person to just sit in front of a piece of paper and talk about what is on their mind. For a digital diary, however, accommodating verbal speech is trivial, opening the diary activity to young people who have less affinity to writing. Furthermore, worksheets accompanying PPIs usually present one way of approaching an activity, for example, providing a table and asking the person to fill it in. Digitally, it is possible to provide a range of options to accommodate differences between young people and allow them to choose. As stipulated by Michel et al [20], there is no technological barrier to replacing writing with audio or video recording and to integrate more interactive modalities into all types of evidence-based PPI. Doing so may help to resolve engagement issues with PP apps for young people. However, we cannot simply take, for example, *Gratitude Journal*; replace its writing components with audio; and assume that its existing evidence base is not impacted by this translation.

The reason why addressing individual preferences and modalities in the context of new digital instantiations of PPI for young people is nontrivial is intimately entangled with one of the most significant gaps in PP research, as has been pointed out from *inside* the field [30] and from *outside* of it [28]: a lack of verified mechanisms of action (MOAs). Ideally, MOA would tell us what aspects of a PPI contribute to its effectiveness, which in turn would allow us to translate the PPI more confidently to best fit the diverse audiences and types of platforms, without the risk of losing the effectiveness of interventions. Unfortunately, most MOAs for PPIs are only speculative [24]. For example, *Gratitude Letter* is the most effective intervention within PP [24]; the activity usually consists of writing a letter to a person you are grateful for and then delivering that letter. However, we do not know if it makes a difference (1) to write the letter by hand or digitally; (2) to deliver it in person or via email; (3) to extend the letter with images, videos, audio recordings, or other multimedia; or (4) to craft the letter entirely as an audio recording or a video log, etc. Although *Gratitude Letter* is a widely used successful intervention, we do not know enough about it to confidently instantiate it digitally without losing its effectiveness.

Overall, to address the issue of young people’s lack of engagement with digital instantiations of intervention strategies, extensive further research is necessary. This includes facilitating the creation of culturally appropriate intervention strategies, exploring risks, and safeguarding strategies relating to PPI and further research into the MOA of the PPI, for example, via microrandomized trials [31]. This will allow us to preserve the evidence base of the established PPI while translating them into engaging, mental health–promoting digital experiences for young people. We have seen young people been able to express their needs and wants very clearly, which further emphasizes the role of co-design workshops as a primary mechanism to create digital interventions [32].

### PP and the Tyranny of the Positive Attitude

Some criticism during the workshop was directed at what our participants felt was too much focus on positive emotions within the PPI they were shown. This criticism mirrors a larger discussion of what has been coined *Tyranny of the Positive Attitude* [33,34], a perception that PP attempts to ignore negative emotions despite their importance to a well-balanced psyche. It is not entirely clear how justified this perception was. In 1990, around the time the modern school of PP was born, Seligman [35] wrote about the importance of negative emotions and the dangers of overly focusing on the positive. In 2016, PP’s (current) *second wave* made an explicit effort to conceptually integrate negative emotions, trauma, suffering, mortality, and adversity into a larger positive framework [36]. Thus, there seems to be some disconnect between the theoretical underpinnings of PP, which long recognize and appreciate the nuanced relevance of negative emotions, and the perception that negative emotions are pushed aside in PP, as in our workshop. A possible explanation could be that our participants responded to individual PPIs, not PP overall. Although the field of PP integrates negative emotions, individual PPI may appear to be overly focused on positive emotions. Our workshop suggested that some young people may respond distinctly negatively to those PPIs. Meanwhile, some PPIs that did have their focus on integrating negative emotions, such as *Healing Through Writing*, were criticized as potentially triggering negative emotions (theme 2). Preference toward one or the other may be a matter of individual differences. We suggest that similar future studies with young people include an explicit discussion of *happiness* to prime aspects of the (presumably) less intuitive notion of eudemonic happiness.

### Limitations

We will now address some limitations of this study, with regard to the role of our setting (ie, a school), our participants (ie, classmates from largely shared environments), and how we exposed them to PPI (ie, through descriptions of activities, not the activities themselves).

This paper is based on one workshop with 30 young people from a specific area in Germany; both the number of participants and their cultural context limit the extent to which our findings generalize. Instead, our data serve as a starting point for future explorations into other groups and cultures of young people.

Our specific setting (ie, in-class workshop) may have been impacted by peer pressure, for example, of not speaking too positively about some kinds of PPI. As the individual ratings from step 1 aligned with opinions expressed during the group discussion in step 2, we do not expect peer pressure to have had a big impact; however, it could have skewed the group discussions. Responses to intervention principles such as kindness, gratitude, and forgiveness, while being around classmates, could have been skewed by teenagers attempting to manage their identity within their social group, for example, trying to conform to gender stereotypes. To alleviate the impact of this, we started with participants responding to PPI individually and in writing, but the setting may have still influenced their responses.

Furthermore, although PP is generally considered useful for anyone regardless of underlying mental health problems, previous experience with mental health problems may skew someone’s opinion toward PP. We did not evaluate the mental health status of our participants. As they all have been from the same class and thus share an environment for long periods, it is possible that shared stress factors may have impacted their attitude as a whole in a way that would not show up in another sample from another school.

In addition, we noticed during the analysis that many of our participants criticized interventions for not taking negative emotions into account. PP overall recognizes the importance of negative emotions, and when we introduced the topic to our participants, we explained this. However, they still evidently felt that the specific interventions they discussed did not sufficiently account for negative emotions. A different selection of interventions with an explicit focus on negative emotions may have resonated better with our participants.

Finally, our participants responded to instructions for PPI, not to first-hand experiences of applying the PPI. It is almost certain that actively applying the PPI would have impacted our participants’ opinions, although it is less certain in which way. For example, when our participants reflected on whether they expected a PPI to help them, their answers were speculative and not necessarily rooted in experience. The attitudes they expressed may not predict if and how they would perceive these interventions in a real-world setting; they might, however, indicate the willingness to try or reject similar intervention strategies outright.

### Conclusions

To make PP useful for young people, we need to find ways to present the interventions as being suitable for the culture and context of young people, be aware of their degree of difficulty, and find ways of aligning interventions with young people’s personalities and strengths. These adjustments seem to be necessary if we expect young people to engage with digital instantiations of PPI principles. Across all our findings, there was a need for customization and choice. It is possible for young people to select and choose which interventions they feel are most useful to them and to further shape these interventions to their own needs, strengths, interests, backgrounds, values, and context in general. Digital technologies are uniquely suitable to facilitate this kind of freedom; but, as past research has shown, they are not yet delivering on it. Future research needs to identify on a more granular level how components of PPI interact with different types of young people’s personalities, interests, and strengths, to allow the design of useful and engaging platforms, which offer young people both the necessary freedom and scaffolding to create individually ideal PPIs. Specifically, we would like to see more research identifying the MOA of interventions and how these mechanisms may be expressed on digital platforms. If these insights would exist, it would become possible to systematically create digital interpretations of interventions, flexible to individual needs and preferences of young people, while preserving their validated effectiveness.

## Appendix

Multimedia Appendix 1 Data showing the relationship between fit, expectation, and inclination, as indicated by the Likert scale rating during step 1 of the research phase.

## Abbreviations

MOA: mechanism of action
PAF: Person-Activity Fit
PP: positive psychology
PPI: positive psychology intervention

## References

[1] McCormick K, McCallion G, Pratt L, Morgan T, Henneman L, Qadri A, Sharma R, Vignoles L (2017). Global Youth Wellbeing Index 2017. International Youth Foundation.

[2] Rickwood DJ, Deane FP, Wilson CJ (2007). When and how do young people seek professional help for mental health problems?. Med J Aust.

[3] Wolfe I, Macfarlane A, Donkin A, Marmot M, Viner R (2014). Why children die: death in infants, children, and young people in the UK. Royal Coll Paediat Child Health.

[4] (2017). The Good Childhood Report. The Children's Society.

[5] Kessler R, Berglund P, Demler O, Jin R, Merikangas K, Walters E (2005). Lifetime prevalence and age-of-onset distributions of DSM-IV disorders in the national comorbidity survey replication. Arch Gen Psychiatry.

[6] Seligman M, Csikszentmihalyi M (2014). Positive psychology: an introduction. Flow and the Foundations of Positive Psychology.

[7] Fearns P (2015). Introduction: the disease model of mental health: a system in crisis. A Prescription for Psychiatry: Why We Need a Whole New Approach to Mental Health and Wellbeing.

[8] Schulz D (2013). A History of Modern Psychology.

[9] Maslow A (2013). Toward a Psychology of Being.

[10] Huppert F (2012). A population approach to positive psychology: the potential for population interventions to promote well-being and prevent disorder. Positive Psychology in Practice.

[11] Bono G, Emmons R, Mccullough M (2012). Gratitude in practice and the practice of gratitude. Positive Psychology in Practice.

[12] Gulliford L, Morgan B (2017). The meaning and valence of gratitude in positive psychology. The Routledge International Handbook of Critical Positive Psychology.

[13] Schueller S (2014). Person–activity fit in positive psychological interventions. The Wiley Blackwell Handbook of Positive Psychological Interventions.

[14] Patel V, Saxena S, Lund C, Thornicroft G, Baingana F, Bolton P, Chisholm D, Collins P, Cooper J, Eaton J, Herrman H, Herzallah M, Huang Y, Jordans M, Kleinman A, Medina-Mora ME, Morgan E, Niaz U, Omigbodun O, Prince M, Rahman A, Saraceno B, Sarkar B, De Silva MD, Singh I, Stein D, Sunkel C, UnÜtzer J (2018). The Lancet Commission on global mental health and sustainable development. Lancet.

[15] (2020). NHS Apps Library. NHS.

[16] (2018). Tools and Apps. Reach Out.

[17] Christensen H, Reynolds J, Griffiths K (2011). The use of e-health applications for anxiety and depression in young people: challenges and solutions. Early Interv Psychiatry.

[18] Frith E (2018). Access and Waiting Time in Children and Young People's Mental Health Services. Education Policy Institute.

[19] Kenny R, Dooley B, Fitzgerald A (2016). Developing mental health mobile apps: exploring adolescents' perspectives. Health Informatics J.

[20] Michel T, Tachtler F, Slovak P, Fitzpatrick G (2019). A Review of Youth Mental Health Promotion Apps Towards Their Fit With Youth Media Preferences. Endorsed Transactions on Pervasive Health and Technology.

[21] Braun V, Clarke V (2006). Using thematic analysis in psychology. Qual Res Psychol.

[22] Mohr D, Schueller S, Riley W, Brown H, Cuijpers P, Duan N, Kwasny M, Stiles-Shields C, Cheung K (2015). Trials of intervention principles: evaluation methods for evolving behavioral intervention technologies. J Med Internet Res.

[23] (2020). The Positive Psychology Toolkit. PositivePsychology.

[24] Parks A (2014). The Wiley Blackwell Handbook of Positive Psychological Interventions.

[25] Dallmayr F (1980). Heidegger on intersubjectivity. Hum Stud.

[26] Nietzsche F (2002). Beyond Good and Evil: Prelude to a Philosophy of the Future.

[27] Fleming T, Merry S, Stasiak K, Hopkins S, Patolo T, Ruru S, Latu M, Shepherd M, Christie G, Goodyear-Smith F (2019). The importance of user segmentation for designing digital therapy for adolescent mental health: findings from scoping processes. JMIR Ment Health.

[28] Wong P, Roy S (2018). Critique of positive psychology and positive interventions. The Routledge International Handbook of Critical Positive Psychology.

[29] Wyman P, Gaudieri P, Schmeelk-Cone K, Cross W, Brown H, Sworts L, West J, Burke K, Nathan J (2009). Emotional triggers and psychopathology associated with suicidal ideation in urban children with elevated aggressive-disruptive behavior. J Abnorm Child Psychol.

[30] Worthington Jr E, Wade N, Hoyt W (2014). Positive psychological interventions for promoting forgiveness: history, present status, and future prospects. The Wiley Blackwell Handbook of Positive Psychological Interventions.

[31] Klasnja P, Hekler E, Shiffman S, Boruvka A, Almirall D, Tewari A, Murphy S (2015). Microrandomized trials: an experimental design for developing just-in-time adaptive interventions. Health Psychol.

[32] Thabrew H, Fleming T, Hetrick S, Merry S (2018). Co-design of ehealth interventions with children and young people. Front Psychiatry.

[33] Held B (2002). The tyranny of the positive attitude in America: observation and speculation. J Clin Psychol.

[34] (2020). The Tyranny of the Positive Attitude.

[35] Seligman M Learned Optimism. Internet Archive.

[36] Lomas T, Ivtzan I (2015). Second wave positive psychology: exploring the positive–negative dialectics of wellbeing. J Happiness Stud.

